# Cytochrome-*c* mediated a bystander response dependent on inducible nitric oxide synthase in irradiated hepatoma cells

**DOI:** 10.1038/bjc.2012.9

**Published:** 2012-01-24

**Authors:** M He, S Ye, R Ren, C Dong, Y Xie, D Yuan, C Shao

**Affiliations:** 1 Institute of Radiation Medicine, Fudan University, No.2094 Xie-Tu Road, Shanghai 200032, China

**Keywords:** ionising radiation, bystander effect, cytochrome-*c*, iNOS, ROS

## Abstract

**Background:**

Radiation-induced bystander effect (RIBE) has important implication in tumour radiotherapy, but the bystander signals are still not well known.

**Methods:**

The role of cytochrome-*c* (cyt-*c*) and free radicals in RIBE on human hepatoma cells HepG2 was investigated by detecting the formation of bystander micronuclei (MN) and the generation of endogenous cyt-*c*, inducible nitric oxide (NO) synthase (iNOS), NO, and reactive oxygen species (ROS) molecules.

**Results:**

When HepG2 cells were cocultured with an equal number of irradiated HepG2 cells, the yield of MN in the nonirradiated bystander cells was increased in a manner depended on radiation dose and cell coculture time, but it was diminished when the cells were treated with cyclosporin A (CsA), an inhibitor of cyt-*c* release. Meanwhile the CsA treatment inhibited radiation-induced NO but not ROS. Both of the depressed bystander effect and NO generation in the CsA-treated cells were reversed when 5 *μ*M cyt-*c* was added in the cell coculture medium. But these exogenous cyt-*c*-mediated overproductions of NO and bystander MN were abolished when the cells were pretreated with *s*-methylisothiourea sulphate, an iNOS inhibitor.

**Conclusion:**

Radiation-induced cyt-*c* has a profound role in regulating bystander response through an iNOS-triggered NO signal but not ROS in HepG2 cells.

Ionising radiation induces DNA damage not only in the directly exposed cells but also in their neighbouring nonirradiated cells, termed as radiation-induced bystander effect (RIBE) that was first reported by Nagasawa and Little (1992). Since then, the research advance in RIBE has significantly impacted on the radiobiological studies and cancer risk evaluation. It has been known that a series of bystander responses, including cell killing (Lewis *et al*, 2001; Schettino *et al*, 2003), chromosomal damage (Lehnert and Goodwin, 1997), genomic instability (Seymour and Mothersill, 1997; Morgan and Sowa, 2007), neoplastic transformation (Sawant *et al*, 2001; Mancuso *et al*, 2008), changes in gene expression (Azzam *et al*, 1998), and DNA methylation (Ilnytskyy *et al*, 2009), can be triggered by the soluble molecules that are released from irradiated cells and affect neighbouring cells via gap junction (Azzam *et al*, 2000; Shao *et al*, 2003b) and/or culture medium (Baskar *et al*, 2007; Dickey *et al*, 2009). Because RIBE has an important implication in radiotherapy, tumour cells have been widely applied for the studies on this phenomenon (Shao *et al*, 2003a; Harada *et al*, 2009). Multiple RIBEs, including cell growth stimulation, DNA damage, and cell death, have been observed in tumour cells *in vitro* (Shao *et al*, 2003a, 2003c; Gow *et al*, 2010). Using mouse model, the bystander responses of internal tumour cells or tissues were also confirmed *in vivo*, and cancer-associated events, such as *p*53 alteration, MMPs activity, and epigenetic change, were proved to be involved in the RIBE (Camphausen *et al*, 2003; Koturbash *et al*, 2007; Lemay *et al*, 2011).

Several bystander signalling molecules, such as free radicals (Narayanan *et al*, 1997; Shao *et al*, 2002; Han *et al*, 2009), proteins (Narayanan *et al*, 1999; Shao *et al*, 2008b), calcium flux (Lyng *et al*, 2002; Shao *et al*, 2006), and hormones (Shao *et al*, 2008a) have been disclosed. Recent studies have shown that cytochrome-*c* (cyt-*c*) is also involved in the RIBE either as a sensor of bystander response (Yang *et al*, 2009) or as a signalling factor transmittable through gap junction (Peixoto *et al*, 2009). Our previous study demonstrated that a *p*53-dependent cyt-*c* release from the mitochondria of irradiated cells had an important role in the regulation of RIBE (He *et al*, 2011). cyt-*c* is an electron transporting protein and belongs to a part of the respiratory chain localised in the inner mitochondrial membrane (Schagger, 2002). Release of cyt-*c* from mitochondria into cytoplasm is a key event of radiation-induced apoptosis (Ogawa *et al*, 2002). However, most of the previous works have paid close attention in the role of cyt-*c* itself in RIBE but not its relationship with other bystander signals. To clarify the signalling pathways underlying the cyt-*c*-mediated bystander response, we detected two principal reactive species, that is, reactive oxygen species (ROS) and nitric oxide (NO), and attempted to determine which could be a downstream signal factor modulated by cyt-*c* in RIBE.

## Materials and methods

### Tumour Cell Line

Human hepatoma HepG2 cells (Shanghai Cell Bank, Chinese Academy of Science, Shanghai, China) were cultured in Dulbecco's modified Eagle's medium (Hyclone Co., Beijing, China) supplemented with 10% fetal bovine serum (PAA Laboratories GmbH, Cölbe, Germany), 100 U ml^−1^ penicillin, 100 U ml^−1^ streptomycin, and 2 mM glutamate. All cell cultures were maintained in a humidified atmosphere of 5% CO_2_ in air at 37 °C.

### Cell irradiation, coculture, and drug treatment

HepG2 cells were seeded onto a 26 × 21 mm^2^ coverslip (1.5 × 10^5^ cells) within a 35-mm dish and allowed to grow overnight for cell attachment. Cells were then irradiated with *γ*-rays generated by a ^137^Cs irradiator (Gammacell-40, Nordion International Inc., Kanata, ON, Canada) at a dose rate of 0.83 Gy min^−1^. Immediately after irradiation, the irradiated cells and nonirradiated cells growing on different coverslips were placed face-to-face with a 3-mm gap and cocultured in a 35-mm dish with fresh medium until further assay.

In some experiments, HepG2 cells were pretreated with 5 *μ*M cyclosporin A (CsA, Sigma Co., St Louis, MO, USA) for 1 h and/or 500 *μ*M *s*-methylisothiourea sulphate (SMT, Sigma Co.) for 10 h before irradiation. CsA is an inhibitor of mitochondrial permeability transition pore (MPTP) and SMT is a highly selective inhibitor of inducible NO synthase (iNOS) (Szabo *et al*, 1994). After irradiation, both drugs were immediately washed with PBS triply. Because CsA was stored (1000 × ) in DMSO, 0.1% DMSO was used as the control of CsA treatment. Some of the other cells were treated with 5 *μ*M exogenous cyt-*c* (Sigma Co.) by adding this reagent into the culture medium immediately after irradiation and persisted until the following measurements of micronuclei (MN), ROS, or NO.

### MN assay

Formation of MN were measured with the cytokinesis block technique that has been widely used to estimate genotoxic damage (Fenech, 2007). Briefly, after irradiation and cell coculture, HepG2 cells were treated with 2 *μ*g ml^−1^ cytochalasin-B (Sigma Co.) for 28 h followed by 0.075 M KCl hypotonic treatment for 3 min and then fixed *in situ* with methanol-acetic acid (9 : 1 v/v) for 20 min. Air-dried cells were stained with 20 *μ*g ml^−1^ acridine orange (Sigma Co.) for 3 min. MN were scored in at least 1000 binucleated cells each sample under a fluorescence microscope (Olympus, Tokyo, Japan). The MN yield, *Y*_MN_, was calculated as the ratio of the number of MN to the number of scored binucleated cells.

### Immunofluorescence localisation of cyt-*c*

Intracellular cyt-*c* was immunocytochemically detected *in situ*. Briefly, HepG2 cells (5 × 10^4^ cells) were grown on a glass coverslip for 24 h before irradiation. 12 h after irradiation, HepG2 cells were washed with PBS and fixed with 4% paraformaldehyde for 20 min then incubated with 3% BSA plus 0.5%Triton X-100 in PBS for 1 h to permeabilise the cells and block nonspecific protein interaction. Subsequently, these cells were incubated overnight with the sheep polyclonal cyt-*c* antibody (Abcam, Cambridge, MA, USA) at a 1 : 100 dilution at 4 °C. After removing the unbound antibody by rinsing with PBS, the cells were incubated with FITC-labeled rabbit anti-sheep IgG (H+L) conjugate (Invitrogen, Carlsbad, CA, USA) at a 1 : 200 dilution for 1 h in dark. Cell nuclei were stained with 100 ng ml^−1^ DAPI (Sigma Co.) for 2 min. The stained cells were then secured with coverslip and sealed with mounting medium (Vector Laboratories, Burlingame, CA, USA). The cell fluorescence image was captured with the MicroPublisher digital camera (QImaging, Surrey, BC, Canada) mounted on a fluorescence microscope (Olympus) and analysed with the Image-Pro Plus software (Media Cybernetics, Inc., Bethesda, MD, USA). Cyt-*c*-released cells (cyt-*c* was diffusely expressed in cytosol) and cyt-*c* concentrated cells (cyt-*c* was concentrated relatively in mitochondria around the nucleus) were respectively counted in 10 randomly chosen fields. Approximately, 100 cells from each sample were analysed for the cyt-*c* distribution assay.

### Western Blot Analysis

After the treatments described above, the culture cells (2 × 10^6^) were harvested and treated with the RIPA lysis (Beyotime Biotechnology, Shanghai, China) containing phosphatase inhibitor cocktail (1 : 100) (Sigma Co.) and phenylmethanesulfonyl fluoride (1 mM) (Sigma Co.) for 5 min on ice. Cell lysate was centrifuged at 12 000 rpm at 4 °C for 5 min. Supernatant was collected and total protein concentration was quantified by the bichinconinic acid protein assay kit (Beyotime Biotechnology). Cell lysate (40 *μ*g protein) was boiled in sodium dodecylsulphate (SDS) buffer for 10 min before electrophoresis on 10% SDS-polyacrylamide gel. After transfer to polyvinylidene fluoride membrane (Millipore, Bedford, MA, USA), the blots were blocked with 5% fat-free dry milk-PBST (PBS containing 0.1% Tween-20) for 1 h at room temperature. The membrane was incubated for 16 h at 4 °C with 1 : 1000 dilution of primary antibodies for iNOS (Cell Signaling Technology, Danvers, MA, USA) and *α*-Tubulin (Beyotime Biotechnology). Blots were washed three times with PBST at 5 min intervals followed by incubation with 1 : 2500 dilution of respective horseradish peroxidase-conjugated secondary antibody (Cell Signaling Technology) for 1 h at room temperature. The transferred proteins were visualised with the ChemiDoc XRS system (Bio-Rad Laboratories, Hercules, CA, USA) using an ECL detection kit (Millipore) and analysed with the Quantity One software (Bio-Rad Laboratories).

### Measurement of intracellular ROS and NO

The intercellular ROS and NO were measured *in situ* by using fluorescence probes of 2′,7′-dichlorofluorescein diacetate (DCFH-DA) and 3-amino,4-aminomethyl -2′,7′-difluorescein diacetate (DAF-FM-DA) (Molecular Probes, Eugene, OR, USA), respectively. Briefly, after irradiation, HepG2 cells seeded on 24-well plates (1 × 10^5^ cells) were treated with 3 *μ*M DCFH-DA or 5 *μ*M DAF-FM-DA for 30 min at 37 °C in dark, then additional dye was washed with PBS and the cells were incubated for an additional 30 min at 37 °C in order for a complete de-esterification of the intracellular diacetates. The fluorescence intensity was then recorded by a microplate reader (Synergy HT, BioTek, Winooski, VT, USA) with an excitation wavelength of 488 nm and an emission wavelength of 525 nm for DCFH or an excitation wavelength of 495 nm and an emission wavelength of 515 nm for DAF-FM. The relative levels of ROS and NO were calculated as the mean fluorescence intensity of irradiated cells compared with the mean intensity of control cells without irradiation.

### Statistical analyses

The data presented as mean±s.e. were obtained from two or three independent experiments with three replicates in each. Comparison is performed by the Student's two-tailed *t*-test, whereas multiple comparisons are carried out using one-way analysis of variance. Statistical significance is acceptable at the level of *P*<0.05. Data are analysed with the software SPSS11.5 (SPSS Inc., Chicago, IL, USA).

## Results

### Radiation-induced direct and bystander damage

**Figure 1:**
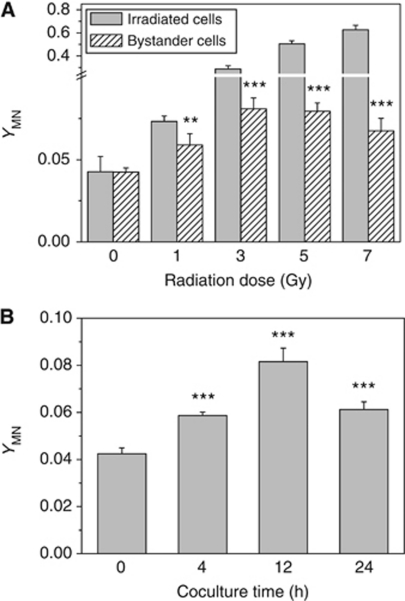
(**A**) Dose response of the yield of MN in the irradiated HepG2 cells and bystander HepG2 cells that were cocultured with irradiated cells for 12 h. (**B**) Time response of bystander MN formation in HepG2 cells that were cocultured with 3 Gy *γ*-irradiated HepG2 cells. ^**^P<0.01, ^***^ *P*<0.001 compared with the control without irradiation.

Figure 1A illustrates that, as a consequence of DNA strand breaks, MN were induced in the irradiated HepG2 cells and its yield increased with dose. When nonirradiated HepG2 cells were cocultured with the irradiated cells for 12 h, MN were induced in the bystander cells. The yield of bystander MN increased when the radiation dose increased to 3 Gy, but then had a tendency of decrease at higher doses even nonsignificantly. The bystander response also had a relationship with the cell coculture time. When the target cells were irradiated with 3 Gy of *γ*-rays, the maximum bystander damage appeared at 12 h during the coculture period from 4 to 24 h (Figure 1B). Accordingly, 12 h post 3 Gy irradiation was an ideal condition for the induction of a bystander response in HepG2 cells and hence was chosen as the representative point for the following mechanistic investigations.

### Influence of cyclosporin-A and cyt-*c* on radiation responses

**Figure 2:**
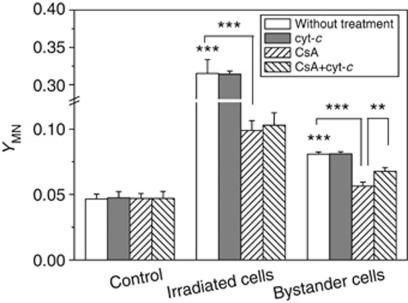
Influence of CsA and exogenous cyt-*c* on the MN formation of 3 Gy *γ*-irradiated HepG2 cells and bystander HepG2 cells, which was cocultured with 3 Gy *γ*-irradiated cells for 12 h. ^**^P<0.01, ^***^P<0.001 compared with the control without irradiation or to the indicated group with drug treatment.

To explore the potential role of mitochondria in radiation damage, 1 h before irradiation, HepG2 cells were treated with CsA to close the mitochondrial membrane pores and inhibit cyt-*c* release. It was found that this treatment not only decreased the yield of radiation-induced MN by about 70% but also significantly diminished the MN formation in the bystander cells (Figure 2), which indicates that the mitochondrial function may be involved in the RIBE.

To obtain more evidence of cyt-*c* being involved in RIBE, exogenous cyt-*c* was added into the medium during coculture of irradiated and bystander cells, and then the MN in both cells were measured. The results showed that although CsA bolcked the induction of bystander MN, when the cells were treated with 5 *μ*M cyt-*c* in substitution for intrinsic cyt-*c* in the CsA-treated cells, the yield of MN in bystander cells was partly recovered from 0.056 of CsA-treated cells to 0.068, but this exogenous cyt-*c* failed to increase the MN yield in the directly irradiated cells under CsA treatment (Figure 2). In addition, this exogenous cyt-*c* treatment itself had no influence on the MN induction of both irradiated and bystander cells. These data suggest that, as a critical event, cyt-*c* could be involved in RIBE rather than direct radiation damage.

### CsA inhibited cyt-*c* release in the irradiated cells

**Figure 3:**
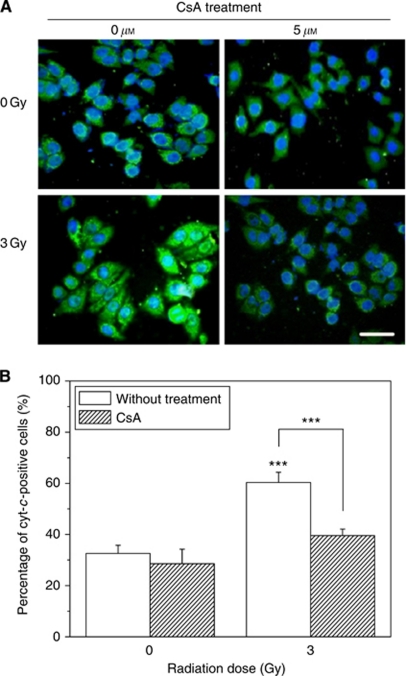
Typical fluorescence image of the distribution of cyt-*c* in HepG2 cells (**A**) and percentage of cyt-*c*-positive cells in the HepG2 population (**B**) 12 h after 3 Gy *γ* irradiation. In some experiments, HepG2 cells were pretreated with 5 *μ*M CsA for 1 h before the irradiation. Green fluorescence indicated cyt-*c*, blue fluorescence indicated nuclei. Scale bar, 20 *μ*m. ^***^P<0.001 compared with the control without irradiation or CsA treatment. The colour reproduction of this figure is available at the *British Journal of Cancer* online.

To know the situation of cyt-*c* release, we measured the distribution of cyt-*c* inside the cytoplasm of HepG2 cells with and without irradiation by the method of immuocytochemistry *in situ*. As shown in Figure 3A, for most of the nonirradiated HepG2 cells, the cellular cyt-*c* protein was concentrated inside mitochondria. After 3 Gy exposure, the cyt-*c* was released from mitochondria and diffused in the whole cytosol so that the percentage of cyt-*c*-released cells was increased from 35% of control to 60%. However, this cyt-*c* release was effectively inhibited by 5 *μ*M CsA so that the percentage of the cyt-*c*-released cells significantly decreased to a level near control (Figure 3B), which is in parallel with the result in Figure 2 that CsA diminishes RIBE on MN induction.

### Generation of radiation-induced NO and ROS

**Figure 4:**
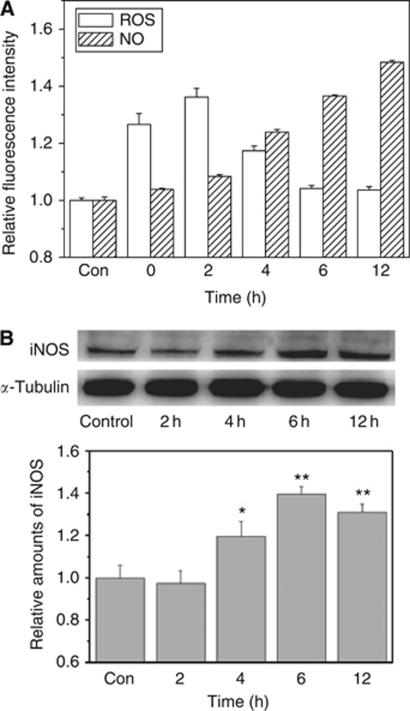
Time course of expression of ROS, NO, and iNOS postirradiation. (**A**) The column plots showed the relative intensity of DCFH and DAF-FM that were calculated as the mean intensity of experimental group cells compared with the mean intensity of control cells. (**B**) The relative expression level of iNOS examined by western analysis that was normalised to *α*-tubulin first and then the ratio of each normalised value to the control value was calculated. ^*^P<0.05, ^**^P<0.01 compared with the control without irradiation.

Two established bystander signalling molecules of NO and ROS were assayed after 3 Gy irradiation. Figure 4A illustrates that the fluorescence intensity of DAF-FM corresponding to the intracellular NO was accumulated in the irradiated HepG2 cells over the time from 2 to 12 h postirradiation and it was significantly increased to about 1.5-fold of the control at 12 h postirradiation. Normally, the intracellular NO is a reaction product of L-arginine catalysed by iNOS. Figure 4B shows that the level of iNOS expression was increased gradually after irradiation and it approached to 1.4-fold of the nonirradiated control at 6 h postirradiation and then became relatively stable up to 12 h after irradiation.

Figure 4A also revealed the time response of radiation-induced ROS that was represented by the relative fluorescence intensity of DCFH in the irradiated cells. It was seen that the kinetics of the intracellular ROS was different from the generation of intracellular NO. ROS was induced immediately after irradiation and its yield increased rapidly to a maximum level at 2 h after irradiation and then decreased gradually to the control level at 4–12 h postirradiation.

### Relationship between radiation-induced cyt-*c* and NO, ROS

**Figure 5:**
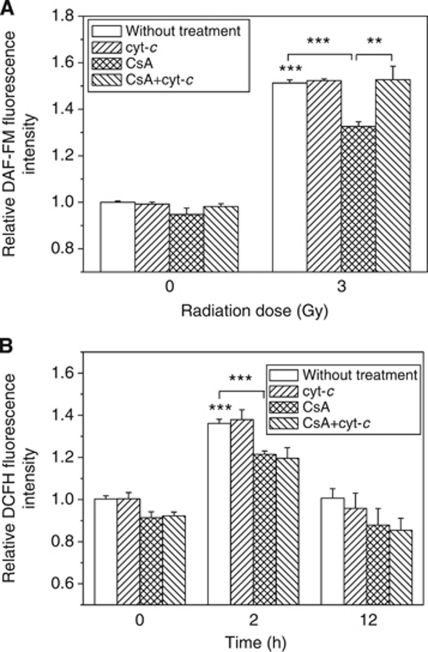
Influence of exogenous cyt-*c* on the induction of NO and ROS in HepG2 cells. (**A**) Relative intensity of NO-induced DAF-FM fluorescence in HepG2 cells 12 h postirradiation. (**B**) Relative intensity of ROS-induced DCFH fluorescence in HepG2 cells 2 and 12 h postirradiation. In some experiments, the irradiated HepG2 cells were pretreated with 5 *μ*M CsA before irradiation. ^***^P<0.001 compared with the control without irradiation and ^**^P<0.01, ^***^P<0.001 between indicated groups.

To know the relationship between cyt-*c* and radiation-induced free radicals, we treated cells with a MPTP inhibitor before irradiation. It was found that when the cells were treated with 5 *μ*M CsA before irradiation, the induction of intracellular NO decreased significantly but still higher than that of nonirradiated control. When the culture medium contained 5 *μ*M cyt-*c* together with CsA, the intracellular level of NO in the irradiated cells was recovered to the level of directly irradiated cells without any drug treatment (Figure 5A). These results disclose that the radiation-induced NO is regulated by the release of mitochondrial cyt-*c*.

More interestingly, although the fluorescence of DCFH in the irradiated cells was effectively reduced when the cells were treated with CsA alone, at the representative time points of 2 and 12 h postirradiation, it was uneventful in the CsA-treated HepG2 and had no relationship to the exogenous cyt-*c* treatment (Figure 5B). Thus, the generation of ROS was not a downstream event of cyt-*c* release. Taken together, our results indicated that NO, rather than ROS, was a downstream product of radiation-induced cyt-*c* release. This finding is coincident with our previous report that NO and ROS were involved in the bystander responses triggered by irradiated tumour cells and normal cells, respectively (Shao *et al*, 2005).

### Relationship between iNOS and cyt-*c* in NO-mediated RIBE

**Figure 6:**
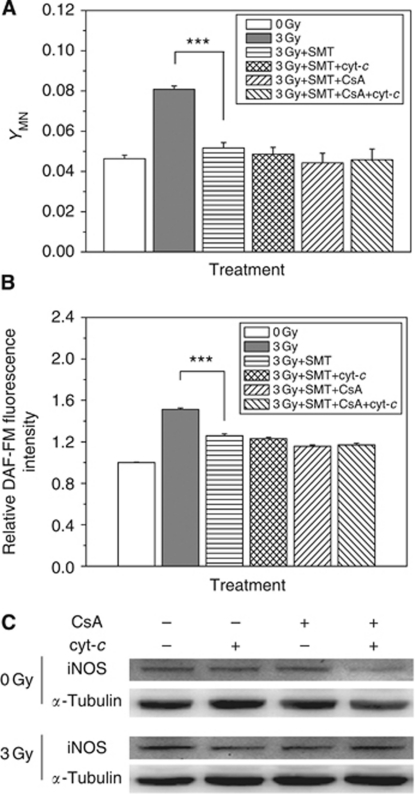
Cyt-*c*-mediated RIBE through NO in an iNOS-dependent pathway. Influence of iNOS inhibitor SMT on radiation-induced bystander MN (**A**) and NO production (**B**). In some experiments, the irradiated HepG2 cells were treated with 5 *μ*M CsA for 1 h before irradiation and/or 5 *μ*M cyt-*c* for 12 h after radiation. ^***^P<0.001 compared with the control without irradiation. (**C**) Effect of CsA and cyt-*c* on the expression of iNOS induced by radiation.

Based on our above results, it can be foreseen that radiation-induced accumulation of iNOS might have a great effect on RIBE. This was confirmed by the result that the treatment of cells with SMT completely suppressed the bystander DNA damage (Figure 6A) and also greatly decreased the level of radiation-induced NO (Figure 6B).

Further experiment was performed to investigate whether iNOS and cyt-*c* are independent of each other in the NO production and its downstream bystander response. Figure 6C illustrates clearly that neither CsA nor exogenous cyt-*c* had any effect on the iNOS expression at 12 h postirradiation, indicating that iNOS was not a downstream molecule of the intracellular cyt-*c*. Considering from the opposite side, we pretreated HepG2 cell with an iNOS inhibitor SMT and then reexamined the cyt-*c*/NO-mediated RIBE. The results showed that although CsA could still somewhat attenuate the radiation-induced NO production, there were no any increases of NO (Figure 6B) and attendant bystander MN in the irradiated HepG2 cells in the presence of exogenous cyt-*c* and iNOS inhibitor (Figure 6A). All of the above results indicated that, as an initial source of NO, the iNOS expression was essential to cyt-*c*/NO-mediated bystander response.

## Discussion

The present study found that the bystander MN could be induced in the nonirradiated HepG2 cells after coculturing with irradiated cells and its yield was dependent on both radiation dose and coculture time, which was consistent with some of the other reports (Harada *et al*, 2008; Asur *et al*, 2009). It can be assumed that the level of bystander response might correspond to radiation-induced cellular damage, but once the cell damage was too serious to be insufficient in generating more bystander signals, the RIBE would reach to a platform and even then descent as that shown in Figure 1A.

RIBE is also related to the cell situation. It was found here that, even at an optimum condition of RIBE, the bystander MN could be nearly eliminated by the treatment of cells with CsA, indicating that mitochondria-dependent intracellular factor(s) may be involved in the stimulation of bystander response. The result of immunocytochemical assay provided a clue that cyt-*c* has a possibility to have a key role in the generation of bystander effect. These conjecture was confirmed by the data that supplement of exogenous cyt-*c* into cell coculture medium partly recovered the bystander MN induction, which had been blocked by CsA, that is, the exogenous cyt-*c* could substitute the function of endogenous cyt-*c* and then stimulates irradiated cells to generate some unknown bystander signalling factors and further induce cellular damage in adjacent cells. However, for the directly irradiated cells, the yield of MN in the CsA-treated HepG2 cells could not be recovered by exogenous cyt-*c*. Accordingly, the cyt-*c* release may be essential for RIBE but is not a key point for direct radiation damage.

Mitochondria is the main pool of radiation-induced cyt-*c* associated with the generation of free radical signals of NO and ROS (Chen *et al*, 2003; Aykin-Burns *et al*, 2011) that could act as important mediators of RIBE (Azzam *et al*, 2004; Shao *et al*, 2008c). NO is postulated to be produced from L-arginine catalysed by iNOS (Nathan, 1992) after irradiation (Matsumoto *et al*, 2001), and it can act as an intercellular signalling molecule, cause DNA damage, and disturb DNA repair in bystander cells (Han *et al*, 2007). Our previous study showed that the amount of NO released from irradiated tumour cells was related to the dose and LET of irradiation, and suggested that radiation-induced NO might be modulated by some unknown factors during cell-programmed death (Shao *et al*, 2001). The present study observed that the generation of NO in the irradiated cells could be significantly inhibited by the treatment of cells with a MPTP inhibitor CsA, but this inhibition could be fully recovered when the exogenous cyt-*c* was supplied to the CsA-treated cells, which confirms that the NO generation is associated with mitochondrial cyt-*c* release in the process of apoptosis.

So far, there are a great deal of interests in the possibility that NO might be generated by a source other than NO synthase, such as the release of *S*-nitrosothiols during deoxygenation (Stamler *et al*, 1997) or the reduction of NO_2_^−^ to NO by haemoglobin (Cosby *et al*, 2003). The precise mechanisms of NOS-regulated intracellular NO and its redox state operated by mitochondrial cyt-*c* were also widely discussed (Torres *et al*, 2000; Palacios-Callender *et al*, 2007). Therefore, it is quite essential and interesting to know the role of iNOS in cyt-*c*-mediated NO generation and bystander effect. We found here that the inhibition of iNOS could suppress the cyt-*c*-mediated RIBE by blocking the source of NO, thus the cyt-*c*/NO-mediated bystander effect was in an iNOS-dependent manner. Based on the present findings we hypothesise that cyt-*c* has an important role in an efficient cascade amplification of the overproduction of NO generated from an iNOS-catalysed reaction.

It is well known that ROS also has an essential role in RIBE (Yang *et al*, 2005) and recent studies make further improvements that mitochondria-dependent ROS was very important in mediating bystander effects (Chen *et al*, 2009). As radiation-induced secondary factors, ROS contributed to the radiation-induced DNA double-strand breaks (Riley, 1994) and could concurrently trigger the release of cyt-*c* from mitochondria in the irradiated cells (Chen *et al*, 2009; Ogura *et al*, 2009). That the ROS level in irradiated HepG2 cells was reduced by CsA but not recalled by exogenous cyt-*c* may be the reason why the bystander MN in the CsA-treated cells was just partly recovered by the exogenous cyt-*c* that could recall the NO level in the CsA-treated cells. An interesting finding here was that the generations of intracellular level of ROS and NO in the irradiated cells had different kinetics. During 12 h postirradiation, ROS increased at first then returned back to a low level, whereas the NO production showed a cumulative effect. It can be proposed that radiation-induced generation of ROS is mainly responsible for direct DNA damage and the early event of bystander effect, meanwhile NO as a persistent bystander signal during cell coculture (Shao *et al*, 2004) is a source of integral process of bystander responses. Taken altogether, our data suggest that the event of radiation-induced cyt-*c* release can modulate NO generation, as a direct bystander signal via an iNOS-dependent mechanism in irradiated hepatoma cells, leading to MN formation in these bystander cells.
